# EFFECT OF CHAIR YOGA ON FRAILTY IN OLDER ADULTS WITH LOWER EXTREMITY OSTEOARTHRITIS: RANDOMIZED CLINICAL TRIAL

**DOI:** 10.1093/geroni/igz038.2527

**Published:** 2019-11-08

**Authors:** Juyoung Park, Zuyun Liu, Edgar R Vieira, Patricia Liehr

**Affiliations:** 1 Florida Atlantic University, Boca Raton, Florida, United States; 2 Department of Pathology, Yale School of Medicine, New Haven, Connecticut, United States; 3 Department of Physical Therapy, Florida International University, Miami, Florida, United States

## Abstract

This study examined whether chair yoga (CY) could reduce severity of frailty in community-dwelling older adults with lower extremity osteoarthritis (OA). Participants were randomly assigned to CY or health education program (HEP) at each of two sites and attended twice-weekly 45-minute sessions for 8 weeks. Data were collected at baseline and 4 and 8 weeks. For primary analysis, followed by Rockwood’s suggesiton, 97 deficits/variables measuring OA symptoms, physical function, balance, fatigue, depression, social activities, and life satisfaction were used to construct a frailty index, ranging from 0 to 1. Fewer deficits/variables were used to construct three alternative versions of the index. Linear mixed-effects models with random intercept were used to analyze longitudinal repeated outcome measures. A total of 112 participants (n = 63 CY, n = 49 HEP; 75.3[7.5] years; 76% female, 46% Hispanic) completed the study. After adjusting for site, cohort effect, and baseline of frailty, there was no significantly greater decline in frailty in the CY group compared to the HEP group (between-group difference, -0.019; 95% CI, -0.063 to 0.025) or the trend of changes in the index (p for interaction = .489). Additional adjustment for baseline characteristics (age, gender, ethnicity, marital status, living alone, health status, pain medication) did not change results substantially. Secondary analysis of three alternative versions of the index indicated similar nonsignificant changes. Thus, an 8-week CY intervention did not reduce severity of frailty in older adults with lower extremity OA. A longer duration of CY with a larger sample size is needed.

